# Impact of NaCl reduction and substitution with KCl and CaCl_2_ on quality attributes of wheat-based bakery products

**DOI:** 10.3389/fnut.2025.1657034

**Published:** 2025-09-22

**Authors:** Daria Musiienko, Lucie Jurkaninová, Diana Chrpová, Lenka Kourimská

**Affiliations:** ^1^Department of Microbiology, Nutrition and Dietetics, Czech University of Life Sciences Prague, Prague, Czechia; ^2^Department of Food Science, Czech University of Life Sciences Prague, Prague, Czechia

**Keywords:** sensory evaluation, food reformulation, bakery products, salt reduction, sodium intake

## Abstract

Excessive sodium intake is a major contributor to hypertension and cardiovascular disease, prompting reformulation of commonly consumed foods such as bakery products. This study investigated the impact of reducing sodium chloride (NaCl) or partially replacing it with potassium chloride (KCl) and calcium chloride (CaCl_2_) on the sensory, rheological, and physical properties of wheat rolls and buns. Eight dough formulations with varied salt content and types were analyzed. Sensory profiling revealed that moderate NaCl reductions (14.4 g/1,000 g) preserved acceptability, while higher reductions or calcium-based substitutions introduced off-flavors such as bitterness and metallic aftertaste. Rheological tests showed that higher NaCl levels enhanced dough stability and consistency, whereas high CaCl_2_ levels decreased stability. Physical evaluation indicated that CaCl_2_ substitution led to higher product volume but also compromised dough cohesion. The findings support the feasibility of moderate sodium reduction and limited substitution in bakery products to improve public health outcomes without sacrificing product quality.

## 1 Introduction

Salt (sodium chloride, NaCl) comprises 40% sodium and 60% chloride and it constitutes approximately 90% of the sodium intake in the human diet (1). It improves taste and has an overall positive effect on various sensory aspects of food. Sodium plays a crucial role in maintaining cellular membrane potential and facilitating nutrient absorption in the small intestine. Additionally, it regulates extracellular fluid volume, thereby sustaining blood volume and blood pressure (2). Despite its essential functions, excessive sodium consumption has been linked to adverse health effects, with elevated blood pressure being the most concerning (3).

Hypertension, defined as blood pressure equal to or exceeding 140/90 mmHg (4), affects over 40 % of the population in many European countries, surpassing 60% in some regions (3). An estimated 1.28 billion adults aged 30–79 years worldwide have hypertension and most (2/3) living in low- and middle–income countries (4). Central and eastern European countries, such as the Czech Republic, Slovenia, and Hungary, exhibit particularly high rates of hypertension, coinciding with their elevated sodium intake (5–7). Hypertension is a major risk factor for cardiovascular disease (CVD), the leading global cause of death. It contributes to 62% of strokes and 49% of coronary heart disease (8).

Numerous prospective cohort studies and outcome trials have highlighted a positive correlation between salt intake and CVD (9). The global mean intake for adults is 4310 mg/day sodium (equivalent to 10.78 g/day salt). This is more than double the World Health Organization recommendation for adults of less than 2,000 mg/day sodium (equivalent to < 5 g/day salt) (10). It has been estimated that reducing average daily salt intake by approximately 5 g, in line with World Health Organization recommendations, could prevent 23% of strokes and 17% of CVD cases (9). Additionally, reducing salt intake has been identified as one of the most cost-effective measures that can be taken to im-prove public health worldwide. It is estimated that reaching a global target of a 30% reduction in salt intake by 2025 (compared to 2010 levels) would save about 40 million lives over 30 years (11).

In industrial countries, total dietary salt intake can be divided into three major sources: approximately 75%−80% comes from the consumption of processed foods, 5%−10% occurs naturally in foods, and 10%−15% is added during cooking or at the table. (2). In contrast, in developing countries, salt used for seasoning plays a much more important role. In China, for example, this accounts for 76% of total salt intake. Soy sauce is also a significant source of sodium in China and Japan (12). Throughout the world, the sodium content of processed foods tends to be many times higher than that of natural foods. An assessment of processed foods in Australia revealed that sauces and spreads contain the most sodium (1,280 mg/100 g) followed by processed meats (850 mg/100 g), snacks (800 mg/100 g), fish products (510 mg/100 g), and bread and bakery products (470 mg/100 g). Food categories low in sodium included cereal and cereal products (210 mg/100 g) and processed fruit and vegetables (210 mg/100 g). The range of sodium content within some categories was extreme, suggesting that it is feasible to produce foods in all categories with lower sodium content (13). A similar study performed in the United Kingdom detected lower sodium levels in bread and bakery products, meat, and sauces and spreads indicating that the sodium content of processed food varies greatly between different countries and likely between different markets (14).

In response to the WHO's global action plan for the prevention and control of non-communicable diseases, several countries, including members of the European Union, have implemented national sodium reduction strategies. For example, the Czech Republic, through its participation in the EU Salt Reduction Framework, has committed to reducing salt intake by encouraging food reformulation, implementing public awareness campaigns, and improving food labeling. These initiatives align with WHO's target of a 30% reduction in mean population salt intake by 2025 (15, 16).

Bhat et al. (17) noted that bakery products serve as the primary source of sodium in various European countries, the USA, and South Africa, constituting 25% to 40% of the total sodium intake in these regions. In South America, bread also emerges as a significant source of sodium, though to a lesser extent, ranging from 15% to 30% in Brazil, Argentina, and Colombia.

Sodium chloride is a major component of most baked goods and is recognized as one of the four essential ingredients of bread, along with flour, water, and yeast. In wheat bread, sodium chloride plays a crucial role in stabilizing gluten, thereby reducing the extensibility of the dough and making it less viscous, ultimately minimizing production losses. Moreover, sodium chloride inhibits yeast fermentation, which leads to a decrease in gas production. When present in lower amounts, sodium chloride accelerates the fermentation rate, causing disproportionate enlargement of the alveoli and compromising the texture of the final product. Additionally, sodium exhibits a hygroscopic property, contributing to a reduction in the water activity of the bread. This, in turn, creates an environment less conducive to microbiological activity, effectively extending the shelf life of the product (18).

Beyond its technological function, bread remains a valuable source of essential nutrients. It provides complex carbohydrates, dietary fiber, B-group vitamins (especially thiamine, niacin, and folic acid), iron, and magnesium. These nutrients are particularly important for low-income populations and children. Therefore, reducing bread intake as a means to lower sodium consumption is not an optimal nutritional strategy. Instead, reformulating bread to reduce its sodium content while preserving its beneficial components represents a more sustainable and health-conscious approach (19, 20).

Girgis et al. (21) found, that gradually reducing the salt content of bread from 2% to 1.5% was possible to achieve without significant differences in the control group, thus confirming that gradual reduction to a certain salt level is feasible. Similar results were found in a study conducted in Holland, where most consumers (85%) were unable to detect a gradual reduction in salt content in bread (22).

Because bakery products are a major source of sodium consumption worldwide salt reduction initiatives should address this food category. Since salt plays a large role in the technical, functional and sensory properties of products, it is necessary to find the best way to reduce salt content without compromising product quality and consumer acceptance. The World Health Organization in the document “World Health Organization Regional Office for Europe nutrient profile model” recommends a maximum 0.5 g of sodium per 100 g of bread and bread products, which corresponds to 1.25 g of table salt.

The sodium content of many foods can be reduced by replacing some proportion of it by potassium chloride. However, this may have a negative effect on some consumers because it leaves a bitter or metallic aftertaste from a certain concentration—unacceptable to the taste (23). Sodium malate also has a salty taste and can be mixed with other salt substitutes. Though containing sodium, its weight percentage is lower (24). In the study conducted by Bassett et al. (25) sodium was replaced with calcium to address widespread calcium deficiency, especially in school-aged children. Theoretical consumption of 50 g baked goods with 50% NaCl replaced by CaCl_2_ aimed to provide 13.5%−17.3% of the recommended daily calcium allowance for children. In another study, a 50% salt substitution with a 1:1 ratio of CaCl_2_ and CaCO_3_ reduced dough ductility, elasticity, and stability. With an initial salt content of 1.8% of flour weight, CaCl_2_ increased top crust hardness and decreased bottom crust hardness. The incorporation of higher proportions of calcium salts resulted in a lighter crust and a more softened interior. Nevertheless, bread with 50% substitution maintained a taste comparable to the control sample with added CaCl_2_ (26).

Bread reformulation is therefore not only a technical challenge but a necessary step to align industrial food production with public health goals. By focusing on a staple food such as bread, sodium reduction efforts can achieve a broader population-level impact, supporting national and international health commitments.

This study aimed to evaluate the effects of reducing NaCl content and partially substituting it with KCl or CaCl_2_ on the sensory, rheological, and physical properties of wheat-based rolls and buns, with the goal of identifying formulations that achieve sodium reduction without compromising product quality or consumer acceptability.

## 2 Materials and methods

Eight dough samples with different inorganic salts: sodium chloride (NaCl, p.a., Lach-Ner s.r.o., Czech Republic), potassium chloride (KCl, p.a., Lach-Ner s.r.o., Czech Republic), and calcium chloride (CaCl_2_, p.a., Lach-Ner s.r.o., Czech Republic) were prepared in Backaldrin s.r.o. Each dough contained 1,000 g of wheat flour T530, 550 g of water, 40 g of fresh yeast (Vivo brand), 40 g of rapeseed oil and 10 g of baking preparation RIP 1% (Backaldrin s.r.o.). This bakery preparation contained wheat flour, emulsifier E481, barley malt extract, ascorbic acid, and enzymes, including diastase (ATC code A09AA01). The salt content in individual samples is seen in Table 1 together with the total weight of dough. Sample 1 was the standard recipe commonly used in bakeries containing 20 g of NaCl per 1,000 g of flour. Samples 2 to 4 were reformulated with reduced NaCl content. Samples 5 and 6 were prepared with a partial of KCl, while samples 7 and 8 were prepared with a replacement of CaCl_2_ to the total 18.0 g of salts similarly to sample 2.

**Table 1:** Salts content in tested samples [in g per 1,000 g of flour] and the final weight of dough [in grams].

**Sample**	**NaCl [g/1,000 g]**	**KCl [g/1,000 g]**	**CaCl_2_ [g/1,000 g]**	**Total weight of dough [g]**
1	20.0	-	-	1660.0
2	18.0	-	-	1658.0
3	14.4	-	-	1654.4
4	12.6	-	-	1652.6
5	14.4	3.6	-	1658.0
6	12.6	5.4	-	1658.0
7	14.4	-	3.6	1658.0
8	12.6	-	5.4	1658.0

Sample codes correspond to specific salt reduction or substitution strategies. Samples 1–4: NaCl only (gradual reduction); samples 5–6: partial substitution of NaCl with KCl; samples 7–8: partial substitution of NaCl with CaCl_2_.

The water for making the dough was preheated to 18 °C. The ingredients were mixed in a dough mixer (Dierks & Söhne GmbH, Germany): first 6 min on low speed (approximately 100 rpm), then another 6 min on high speed (approximately 200 rpm). After mixing, the temperature of the kneaded dough was checked, which was then matured for 40 min. After ripening, the dough was divided into equal parts. Two-thirds of these portions were transferred to the dough molder (Universum 50/70 Artos, Czech Republic), where they were formed into rolls, while one-third was shaped in the form of buns. The semi-finished products were placed in a 30 °C oven to rise for 40 min, and then baked in the oven (MIWE Michael Wenz GmbH, Germany) for 12 min at a set temperature of 220 °C.

### 2.1 Sensory analysis

The sensory analysis involved 43 semi-trained assessors who had received basic training before the assessment. They were 22 men and 21 women of the age from 19 to 74 years. A sensory profiling method using a 100mm graphical linear oriented unstructured scale was applied. The rated descriptors and scale orientations are shown in Table 2.

**Table 2:** Sensory descriptors and scales their orientation.

**Descriptor**	**Scale orientation**
Flavor intensity	
Sweet taste intensity	
Salty taste intensity	
Bitter taste intensity	0% - imperceptible; 100% - very intensive
Metallic taste intensity	
Soapy taste intensity	
Aftertaste intensity	
Pleasantness of texture	0% - disgusting; 100% – excellent
Overall flavor pleasantness	
Cohesion	0% - very crumbly; 100% - very cohesive

### 2.2 Measurement of flour properties and baking test

Eight wheat flour doughs described in Table 1 were subjected to analysis by a farinograph for the systematic investigation of their rheological properties. Conforming to ISO standard 5530-1:1988, each dough formulation maintained a constant flour quantity of 300 g. The farinograph characteristics of the wheat flour and the flour blends, including water absorption (corrected to 500 BU), dough development time, dough stability, and mixing tolerance index (MTI), were determined according to ICC Standard No. 115/1 (27) using a Brabender Farinograph (SEW, No. 1102391; Brabender, Duisburg, Germany).

The next step involved monitoring of the weight and other parameters of the baked samples. Individual samples were weighed three times with 30-min intervals, with the first weighing conducted 30 min after removal from the oven. The final measurement confirmed the stabilization of weight when the samples were completely cooled down. Samples were weighed in three sets, where one set consisted of 3–5 randomly selected buns and two sets each consisted of five rolls. The weighing was done with a precision of three decimal places.

After cooling for 90 min, the specific volume of the baked products was determined according to AACC Method 10-05.01, using the rapeseed displacement method as described by Whitaker and Barringer (28). The dimensions of the samples were measured manually using a ruler between two fixed points corresponding to the maximum extension of the baked item. For rolls, length, width, and height were recorded; for buns, only width and height were measured. Each dimensional parameter was measured on five replicates for rolls and three for buns. The arithmetic mean of replicate measurements was calculated to obtain representative values for statistical evaluation.

### 2.3 Statistical analysis

Statistica 12 software (StatSoft GmbH., Hamburg, Germany) was used to process the obtained data. All correlations and differences between samples were considered statistically significant at the chosen alpha level 0.050.

Cluster analysis (using the Euclidean distance) was conducted to assess the inter-similarity of samples, aiming to ascertain the optimal salt or salt combinations as substitutes for NaCl based on their likeness to the original recipe of the baked goods. The analysis included the study of organoleptic and rheological properties of the dough as well as related physical characteristics, considering all these three dimensions simultaneously.

The Kruskal–Wallis rank sum test was used to evaluate statistically significant differences between independent groups for sensory evaluation. When significant differences were found (*p* < 0.05), *post-hoc* pairwise comparisons were conducted using Dunn's test, with *p*-values adjusted by the Benjamini–Hochberg FDR method. Statistical analysis for rheological characteristics of dough and physical parameters of baked products was performed using one-way analysis of variance (ANOVA) followed by Tukey's HSD *post-hoc* test for multiple comparisons. Results are expressed as means ± standard deviation (*N* = 3). Different superscript letters within a column denote statistically significant differences between formulations at *p* < 0.05.

## 3 Results

Results from sensory analysis, dough properties, and bakery products volume and dimensions parameters were first evaluated separately, and then their correlations were assessed.

### 3.1 Sensory analysis results

Table 3 presents the arithmetic mean and standard deviation of the sensory profile evaluation of baked rolls and buns with different salt contents. The samples were selected randomly, and the assessors evaluated both the crumb and crust of the products, acknowledging potential differences in taste perception due to the Maillard reaction's influence in the crust.

**Table 3:** Sensory analysis of rolls and buns with different content and types of salts.

**Parameter [% of scale]**	**1**	**2**	**3**	**4**	**5**	**6**	**7**	**8**
Texture pleasantness	56 ± 18	60 ± 19	57 ± 18	53 ± 21	55 ± 17	54 ± 17	55 ± 17	49 ± 20
Cohesion	65 ± 18	62 ± 17	55 ± 18	55 ± 18	60 ± 17	64 ± 18	56 ± 19	56 ± 22
Overall flavor pleasantness	58 ± 19	57 ± 18	54 ± 18	45 ± 18	53 ± 17	53 ± 18	47 ± 19	40 ± 22
Flavor intensity	53 ± 22	50 ± 20	42 ± 19	40 ± 19	46 ± 14	49 ± 17	43 ± 17	45 ± 20
Sweet taste intensity	30 ± 24	30 ± 21	32 ± 23	27 ± 21	26 ± 20	29 ± 23	28 ± 20	20 ± 19
Salty taste intensity	42 ± 24	39 ± 21	27 ± 18	28 ± 17	31 ± 22	27 ± 19	31 ± 22	27 ± 20
Bitter taste intensity	18 ± 23	17 ± 18	16 ± 18	15 ± 17	22 ± 22	14 ± 17	20 ± 20	29 ± 27
Metallic taste intensity	15 ± 21	13 ± 19	13 ± 15	9 ± 10	17 ± 21	10 ± 13	16 ± 18	23 ± 24
Soapy taste intensity	13 ± 18	16 ± 21	14 ± 18	10 ± 15	14 ± 18	12 ± 15	15 ± 19	22 ± 26
Aftertaste intensity	20 ± 22	21 ± 23	22 ± 21	18 ± 17	23 ± 23	15 ± 18	24 ± 23	37 ± 32

Results are expressed as arithmetic mean ± standard deviation.

Significant differences were identified for overall flavor pleasantness, salty taste intensity, and aftertaste intensity.

For overall flavor pleasantness, sample 8 consistently received lower acceptability scores, showing significant differences compared to samples 1 (*p* = 0.0004), 2 (*p* = 0.0008), 3 (*p* = 0.0071), 5 (*p* = 0.0114), and 6 (*p* = 0.0082). Sample 1 also differed significantly from samples 4 (*p* = 0.0071) and 7 (*p* = 0.0186), and sample 2 from sample 4 (*p* = 0.0114) and sample 7 (*p* = 0.0405). The results show that reducing NaCl content in the dough to the level of sample 3 (14.4 g/1,000 g) does not cause a significant decrease in overall flavor pleasantness. However, further reduction of salt, especially when partially replaced by CaCl_2_ as in sample 8 (12.6 g NaCl + 5.4 g CaCl_2_), significantly lowers flavor acceptability. Therefore, salt content can be safely reduced to the level of sample 3 without noticeable loss in taste.

For salty taste intensity, sample 1 was perceived as significantly saltier than samples 3 (*p* = 0.0233), 4 (*p* = 0.0254), 6 (*p* = 0.0233), 7 (*p* = 0.0480), and 8 (*p* = 0.0233). Additionally, samples 6 and 8 differed significantly from sample 2 (*p* = 0.0480 for both), indicating clear variation in perceived saltiness among the formulations. However, reducing salt content to the level of sample 2 or using formulations like sample 5 did not result in significant differences in perceived saltiness compared to sample 1. Therefore, salt content can be safely lowered to the levels used in samples 2 (18 g/1,000 g) or 5 (14.4 g NaCl + 3.6 g KCl) without noticeable loss of saltiness, while further reductions lead to significant changes in taste.

The significant difference in aftertaste intensity observed only between samples 6 and 8 (*p* = 0.0148), with sample 8 exhibiting the strongest aftertaste, suggests that the addition of CaCl_2_ has a more pronounced impact on aftertaste than KCl at comparable levels. This indicates that CaCl_2_ at 5.4 g/1,000 g may contribute to a stronger and potentially less desirable aftertaste when used as a partial salt replacer.

Concerning correlations among various sensory parameters there was a medium positive correlation between overall taste pleasantness and overall texture pleasantness (correlation coefficient r = 0.6147). Furthermore, a significant weak positive correlation was identified between overall taste intensity and intensity of salty taste (r = 0.3990).

Interesting were medium correlations among bitter, metallic, and soapy taste intensities with aftertaste intensity. These categories exhibit clear direct proportional relationships, as evidenced by the correlation of aftertaste intensity with bitter (r = 0.5671), metallic (r = 0.5341), and soapy (r = 0.5438) tastes. Additionally, a similar relationship was observed between metallic and bitter tastes intensities (correlation coefficient r = 0.6616).

**Figure 1:**
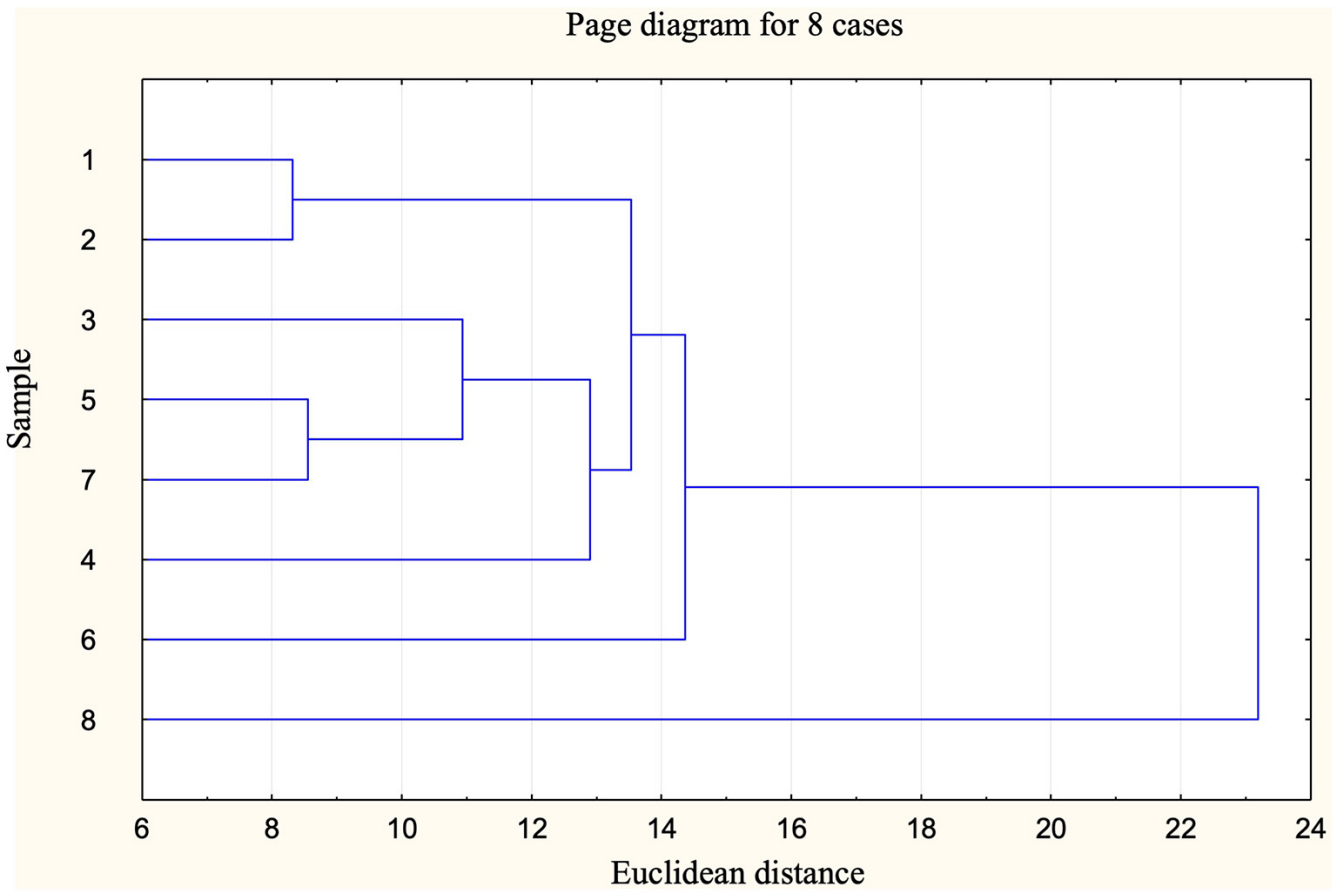
Single linkage cluster diagram of samples according to their sensory properties.

The cluster analysis (Figure 1) conducted on all samples using all sensory parameters indicates a notable similarity between samples 6 and 8 (samples with higher replacement of NaCl by other salts). Additionally, sample 4 with the lowest content of NaCl exhibits a degree of similarity to these two, albeit to a slightly lesser extent.

### 3.2 Rheological and physical results

The effect of varying salt content and type on the rheological properties of wheat flour dough was evaluated (Table 4). Water absorption ranged narrowly between 61.4% and 61.8% across all samples. The highest water absorption was observed in sample 2 (61.8%), while the lowest values (61.4%) were recorded for samples 4, 6, 7, and 8.

**Table 4:** Rheological characteristics of dough and physical parameters of baked products made with different contents and types of salts.

**Sample**	**Water absorption [%]**	**Dough development time [min]**	**Dough stability time [min]**	**Degree of softening [BU]**
1	61.7 ± 0.23^a^	7.75 ± 0.13^c^	19.25 ± 0.40^d^	30 ± 1.28^a^
2	61.8 ± 0.23^a^	6.00 ± 0.13^a^	13.25 ± 0.40b^c^	30 ± 1.28^a^
3	61.5 ± 0.23^a^	8.00 ± 0.13^cd^	12.25 ± 0.37^ab^	40 ± 1.71^b^
4	61.4 ± 0.23^a^	6.25 ± 0.13^a^	13.50 ± 0.41^bc^	40 ± 1.71^b^
5	61.6 ± 0.23^a^	8.50 ± 0.13^d^	14.75 ± 0.44^c^	30 ± 1.28^a^
6	61.4 ± 0.23^a^	7.75 ± 0.13^c^	13.75 ± 0.41^bc^	40 ± 1.71^b^
7	61.4 ± 0.23^a^	7.50 ± 0.13^bc^	10.50 ± 0.32^a^	40 ± 1.71^b^
8	61.4 ± 0.23^a^	7.00 ± 0.13^b^	11.75 ± 0.35^ab^	40 ± 1.71^b^

Values are presented as means ± standard deviation (*N* = 3). Different letters within a column indicate statistically significant differences among formulations (*p* < 0.05). BU, Brabender Units.

Dough development time varied between 6.00 and 8.50 min. The shortest development time was observed in the sample 2 (6.00 min), while the longest was in the sample 5 (8.50 min). Samples 3, 5, and 6 showed relatively higher development times compared to the others.

Dough stability, defined as the duration the dough maintains its optimal consistency, ranged from 10.5 to 19.25 min. The longest stability was found in the control sample (1) with 19.25 min, while the lowest was observed in sample 7 (10.5 min). Samples 2, 3, 4, and 8 had intermediate values between 11.75 and 13.5 min.

The degree of softening, expressed in Brabender Units (BU), was 30 BU in samples 1, 2, and 5, indicating firmer dough structure, while samples 3, 4, 6, 7, and 8 exhibited higher softening values of 40 BU.

Physical parameters of buns made from the different doughs are shown in Table 5. The volume of buns ranged from 197 to 263 cm3. Sample 8 had the largest volume (263 cm3), and sample 6 the smallest (197 cm3). Volumes for samples 1–4 were between 223 and 243 cm3.

**Table 5:** Physical parameters of buns made with different contents and types of salts.

**Buns**	**Bun volume [cm3]**	**Bun height [mm]**	**Bun width [mm]**	**Bun height/width ratio**
1	233 ± 12^a^	54.75 ± 0.43^a^	86.25 ± 0.83^a^	0.64 ± 0.01^a^
2	223 ± 14^a^	54.00 ± 1.41^a^	86.25 ± 0.83^a^	0.63 ± 0.02^a^
3	240 ± 12^a^	53.75 ± 1.09^a^	90.00 ± 1.41^ab^	0.60 ± 0.01^a^
4	243 ± 15^a^	52.75 ± 0.83^a^	91.75 ± 0.43^b^	0.58 ± 0.01^a^
5	217 ± 13^a^	50.25 ± 1.09^a^	88.25 ± 0.43^ab^	0.57 ± 0.01^a^
6	197 ± 12^a^	51.00 ± 3.74^a^	91.00 ± 0.70a^b^	0.56 ± 0.04^a^
7	247 ± 14^a^	50.00 ± 0.71^a^	92.25 ± 1.64^b^	0.54 ± 0.01^a^
8	263 ± 15^a^	54.00 ± 0.71^a^	92.25 ± 0.83^b^	0.59 ± 0.01^a^

Values are presented as means ± standard deviation (N = 3). Different letters within a column indicate statistically significant differences among formulations (*p* < 0.05).

Bun width varied from 86.25 to 92.25 mm. Samples 3 to 8 generally showed greater width than the control. The height ranged from 50.00 mm (sample 7) to 54.75 mm (sample 1). The height-to-width ratio ranged between 0.54 and 0.64, with sample 1 having the highest and sample 7 the lowest ratio.

The physical characteristics of rolls are presented in Table 6. Roll volume varied from 206 cm3 (sample 1) to 266 cm3 (sample 8). Intermediate values were observed in samples 3–7 (216–250 cm3). Roll width ranged from 52.25 to 57.33 mm, with sample 8 exhibiting the widest roll. Height varied from 40.67 mm (sample 6) to 48.17 mm (sample 8). The height-to-width ratio ranged from 0.77 to 0.87, with sample 3 achieving the highest ratio.

**Table 6:** Physical parameters of rolls made with different contents and types of salts.

**Rolls**	**Roll volume [cm3]**	**Roll height [mm]**	**Roll width [mm]**	**Roll height/width ratio**
1	206 ± 14^a^	43.50 ± 2.90^a^	52.25 ± 2.35^a^	0.84 ± 0.06^a^
2	214 ± 12^a^	41.25 ± 0.92^a^	53.17 ± 1.28^a^	0.78 ± 0.02^a^
3	244 ± 16^a^	46.33 ± 1.18^a^	53.33 ± 0.94^a^	0.87 ± 0.03^a^
4	250 ± 15^a^	44.75 ± 0.92^a^	55.92 ± 1.19^a^	0.80 ± 0.02^a^
5	216 ± 16^a^	44.58 ± 1.11^a^	54.08 ± 1.85^a^	0.82 ± 0.02^a^
6	234 ± 15^a^	40.67 ± 1.03^a^	53.00 ± 1.87^a^	0.77 ± 0.03^a^
7	249 ± 12^a^	44.00 ± 2.20^a^	55.83 ± 2.07^a^	0.79 ± 0.04^a^
8	266 ± 13^a^	48.17 ± 2.82^a^	57.33 ± 2.21^a^	0.84 ± 0.03^a^

Values are presented as means ± standard deviation (*N* = 3). Different letters within a column indicate statistically significant differences among formulations (*p* < 0.05).

### 3.3 Correlations between tested parameters

Statistically significant correlations were observed among physical and rheological descriptors as well as between these descriptors and sensory properties. Strong positive correlations were between the intensity of salty taste and the ratio of height and width of buns (r = 0.7189) as well as between the intensity of salty taste and dough viscosity (r = 0.8543). Negative strong correlation was between the intensity of salty taste and consistency (r = −0.8283).

Correlations between the tested rheological and physical parameters were also separately calculated for the series of the first four samples, where only the NaCl content was reduced (there was no substitution with other salts) (Table 7). There was a negative correlation between the amount of salt and the volume of buns (r = −0.75) and rolls (r = −0.99) indicating a decrease in the volume with an increase in the amount of salt. The height to width ratio of buns shows a very strong positive correlation (r = 0.99) indicating its increase with increasing salt content. Flour binding also correlates positively (r = 0.89) with the increasing NaCl content. Dough stability and increasing salt content has a moderate positive correlation (r = 0.73). Finally, dough resistance shows a high negative correlation (r = −0.95) with increasing salt content.

**Table 7:** Pearson correlation coefficients between rheological characteristics of dough and physical parameters of baked products with reduced NaCl content.

**Parameters**	**Amount of salt**	**Buns – volume**	**Buns – height: width**	**Rolls – volume**	**Binding of flour [%]**	**Dough rise [min]**	**Dough stability [min]**	**Dough resistance [BU]**
Amount of salt	1.00	−0.75	0.99	−0.99	0.89	0.19	0.73	−0.95
Buns – volume	−0.75	1.00	−0.78	0.81	−0.97	0.34	−0.15	0.87
Buns – height: width	0.99	−0.78	1.00	−0.97	0.92	0.23	0.64	−0.92
Rolls – volume	−0.99	0.81	−0.97	1.00	−0.92	−0.05	−0.70	0.98
Binding of flour [%]	0.89	−0.97	0.92	−0.92	1.00	−0.13	0.37	−0.95
Dough rise [min]	0.19	0.34	0.23	−0.05	−0.13	1.00	0.34	0.14
Dough stability [min]	0.73	−0.15	0.64	−0.70	0.37	0.34	1.00	−0.61
Dough resistance [BU]	−0.95	0.87	−0.92	0.98	−0.95	0.14	−0.61	1.00

Strong correlations (|r| ≥ 0.90) are highlighted in red for clarity.

## 4 Discussion

Reformulating bakery products by reducing sodium chloride (NaCl) or replacing it with alternative salts is an important strategy for improving public health. However, maintaining product quality and sensory appeal while reducing salt content on an industrial scale remains a major challenge (29). This study presents a detailed evaluation of the sensory, rheological, and physical impacts of salt reduction and substitution in bread products.

### 4.1 Sensory evaluation

Reducing sodium intake remains a key public health priority to combat hypertension and cardiovascular disease (3, 16). As bread is one of the primary sources of dietary salt in many countries (12, 19), reformulating bakery products presents a practical approach for sodium reduction on a population scale (2, 14). This study examined the impact of stepwise sodium chloride (NaCl) reduction and its partial replacement with potassium chloride (KCl) and calcium chloride (CaCl_2_) on the sensory characteristics of bread rolls.

The results demonstrated that sample 8, containing 12.6 g/1,000 g NaCl and 5.4 g/1,000 g CaCl_2_, received the lowest scores for overall flavor pleasantness. This aligns with earlier findings suggesting that CaCl_2_ negatively impacts flavor perception when used in higher concentrations (11, 25). In contrast, sample 3 (14.4 g NaCl) did not differ significantly from the control (sample 1), indicating that moderate NaCl reduction can be achieved without a loss in acceptability. This finding supports previous studies such as Girgis et al. (21), who showed that a 25% salt reduction in bread may go undetected by consumers.

Hence, reducing NaCl to 14.4 g/1,000 g appears viable without compromising sensory appeal. However, the addition of CaCl_2_ at 5.4 g/1,000 g significantly deteriorates flavor perception, despite its nutritional potential as a calcium fortifier (25, 30).

As expected, sample 1 (20 g NaCl) was perceived as the saltiest. However, samples 2 (18 g NaCl) and 5 (14.4 g NaCl + 3.6 g KCl) did not significantly differ from the control in saltiness perception, suggesting that KCl can effectively compensate for moderate NaCl reduction. These findings align with the results of Gusmão et al. (31) and Dunteman et al. (29), which demonstrated that KCl substitution up to 30% can preserve the salty taste in bread.

In contrast, samples with more aggressive NaCl reduction (samples 3, 4) or with CaCl_2_ substitution (sample 8) were rated significantly less salty, reflecting the diminished sodium signal that contributes critically to overall flavor perception (18, 32).

A significant difference in aftertaste intensity was observed between samples 6 (containing KCl) and 8 (containing CaCl_2_), with the latter producing a distinctly stronger and less pleasant aftertaste. Correlation analysis further indicated that this aftertaste was closely linked to increased perceptions of bitterness, metallic, and soapy notes. These sensory impressions are consistent with previous findings by Bassett et al. (25), who reported that calcium fortification in bread formulations may negatively impact flavor acceptability due to the characteristic taste of calcium salts. Similarly, Salinas et al. (30) highlighted that calcium compounds can interact with gluten proteins and influence not only dough rheology but also the final sensory quality, often contributing to off-flavors. While KCl has also been associated with a slightly bitter or metallic profile (31), our findings suggest that CaCl_2_ impairs sensory acceptability to a greater extent, likely due to its more intense aftertaste and unpleasant flavor notes.

These results underscore the importance of balancing functional benefits of salt substitutes with their sensory impact when reformulating bread products.

Beyond reduced saltiness, samples with higher substitution levels (6–8) showed increased intensities of bitter, metallic, and soapy tastes. These undesirable sensory attributes are commonly associated with elevated concentrations of KCl and especially CaCl_2_, as previously reported by Bassett et al. (25) and Gusmão et al. (31). CaCl_2_ appears to have a more pronounced negative impact, likely due to its higher ionic strength and its interactions with flavor receptors as well as gluten proteins, as described by Qu et al. (33). This may explain the stronger aftertaste and off-flavor perception in samples containing CaCl_2_.

These findings highlight the importance of carefully controlling the dosage of salt substitutes and considering the use of flavor modulators or masking agents to maintain the sensory acceptability of reduced-sodium bread formulations.

Although no statistically significant differences were found among samples regarding texture pleasantness, a moderate positive correlation was observed between texture and flavor acceptability. This supports the idea that favorable textural attributes may help offset negative taste perceptions. Studies such as Beck et al. (32) and Wang et al. (34) have shown that sodium and its substitutes influence dough rheology and structure, which in turn affect the sensory profile of baked products. Moreover, Salinas et al. (30) demonstrated that calcium-based fortification alters dough structure in ways that can affect overall mouthfeel and flavor perception.

Cluster analysis showed clear grouping of samples 6 and 8, both of which had high levels of NaCl replacement, indicating a distinct sensory profile. Sample 4 (lowest NaCl, no substitutes) showed partial similarity, suggesting that either a drastic salt reduction or its substitution notably alters the overall sensory impression. These findings echo conclusions by Antúnez et al. (35) and Cashman et al. (36), who emphasize the need to stay within a perceptual threshold to ensure consumer acceptance.

### 4.2 Rheological properties

This study confirms that NaCl plays a crucial role in modulating the rheological behavior of wheat-based dough. Higher NaCl concentrations enhance dough development time and stability, primarily by promoting gluten network formation, which is a finding in line with previous research (34, 37).

A stepwise reduction in NaCl without substitution led to a noticeable decline in dough stability, likely due to the reduced ionic strength weakening the gluten matrix. Incorporating KCl or CaCl_2_ partially mitigated this loss, though their effects varied. KCl maintained moderate dough stability at low inclusion levels, but at higher levels, it appeared to weaken the gluten structure. In contrast, CaCl_2_ had a more pronounced impact on dough cohesion, significantly reducing stability. This is likely due to the dual role of calcium ions enhancing hydrophobic interactions and elasticity but also disrupting the optimal gluten balance (30, 33).

Although water absorption remained relatively unchanged across samples, salt composition significantly influenced dough development time and stability. The presence of NaCl improved rheological performance by altering protein charge distribution and facilitating gluten cross-linking. These effects are particularly advantageous for leavened products, where a dense, elastic gluten network enhances volume and structural integrity (32, 38).

### 4.3 Physical properties of baked products

The rheological changes were reflected in the physical characteristics of the final baked goods. Reducing NaCl led to slight decreases in volume and height for buns and rolls. Substitution with KCl caused a more substantial decline, likely due to impaired gas retention and gluten integrity. Conversely, CaCl_2_ substitution improved both volume and shape retention, indicating its favorable influence on dough aeration and product morphology.

The highest product volumes were observed in samples with reduced NaCl supplemented by CaCl_2_, supporting the hypothesis that calcium enhances gas retention during fermentation. In contrast, samples with KCl exhibited increased lateral expansion and reduced height, indicating a softer and less elastic dough matrix. These findings align with previous reports that suggest KCl provides limited structural benefits and may compromise texture at higher concentrations (31).

The flour used in this study had high strength, as evidenced by water absorption above 61%, dough development times exceeding 6 min, and stability values greater than 12.25 min. This strong viscoelastic profile likely made the dough less sensitive to changes in salt composition. Therefore, the technological impacts of salt reduction or substitution could be more pronounced when using medium- or low-strength flours, which are generally more susceptible to changes in gluten structure (39).

### 4.4 Technological implications

Although overall farinographic changes were modest, the trends clearly demonstrate that both the type and amount of salt exert measurable effects on dough behavior. NaCl reduction alters gluten development and viscoelastic properties that are critical for optimal processing conditions. Furthermore, the technological response to salt modification appears to depend strongly on flour quality (40).

### 4.5 Practical recommendations

Based on the findings, calcium chloride is a more suitable partial replacement for sodium chloride than potassium chloride, particularly in terms of maintaining dough strength and final product volume. While reducing sodium intake remains a public health priority, achieving this goal must not compromise product quality. Strategic selection and precise dosing of salt alternatives offer a viable approach to meet both nutritional and technological objectives.

Cluster analysis of rheological and physical data grouped samples based on salt content and type, highlighting the influence of both factors on product characteristics. Samples with partial CaCl_2_ substitution (samples 7 and 8) were most distinct, particularly at higher substitution levels. In contrast, the sample with moderate KCl inclusion (sample 5) remained closer to the traditional NaCl formulation.

## 5 Conclusion

This study suggests that NaCl can be reduced to 14.4 g/1,000 g without compromising sensory quality. Furthermore, partial replacement with KCl up to 3.6 g/1,000 g can maintain saltiness without introducing strong off-flavors. However, CaCl_2_ at 5.4 g/1,000 g significantly impairs flavor and should only be used in small amounts or with masking strategies.

Given the global initiatives to reduce salt in processed foods and national reformulation strategies, our findings support the feasibility of balanced approaches: moderate salt reduction, selective use of substitutes, and optimization of dough characteristics.

From a rheological perspective, NaCl was shown to enhance dough development time, stability, and structure by promoting gluten network formation. Its reduction led to decreased dough stability and increased softening, especially in samples without substitution. The introduction of KCl partially compensated for these changes: at lower substitution levels, it preserved acceptable dough development and consistency, but higher KCl levels resulted in weakened dough structure and lower product volume. This was likely due to KCl's limited ability to support gluten network strength compared to NaCl.

In contrast, CaCl_2_ substitution increased product volume and improved aeration but negatively impacted sensory qualities, introducing bitterness and metallic aftertastes. These effects were particularly pronounced at higher inclusion levels. KCl, while less disruptive to flavor than CaCl_2_, still affected dough rheology and should be used cautiously in formulations targeting both structural integrity and sensory acceptability.

Overall, successful reformulation requires a careful balance between sodium reduction and maintenance of sensory and rheological quality. Future research should explore optimized combinations of salt substitutes, evaluate consumer preferences across different demographics, and consider flour quality as a critical factor influencing the outcome of sodium reduction strategies in baked goods.

## Data Availability

The original contributions presented in the study are included in the article/supplementary material, further inquiries can be directed to the corresponding author/s.
